# Changing face of Candida colonization pattern in pediatric patients with hematological malignancy during repeated hospitalizations, results of a prospective observational study (2016–2017) in shiraz, Iran

**DOI:** 10.1186/s12879-019-4372-x

**Published:** 2019-08-30

**Authors:** Seyedeh Sedigheh Hamzavi, Ali Amanati, Parisa Badiee, Mohammad Rahim Kadivar, Hadis Jafarian, Fatemeh Ghasemi, Sezaneh Haghpanah, Mansooreh Dehghani, Abbas Norouzian Baghani

**Affiliations:** 1 Professor Alborzi Clinical Microbiology Research Center, Shiraz University of Medical Sciences, Shiraz, Iran; 2 Head of Infection Control Unit, Amir Medical Oncology Hospital, Shiraz University of Medical Sciences, Shiraz, Iran; 3 Hematology Research Center, Shiraz University of Medical Sciences, Shiraz, Iran; 4 Department of Environmental Health Engineering, School of Health, Shiraz University of Medical Sciences, Shiraz, Iran; 5 Department of Environmental Health Engineering, School of Public Health, Tehran University of Medical Sciences, Tehran, Iran

**Keywords:** *Candida albicans*, Fungal colonization, Pediatric leukemia

## Abstract

**Background:**

Surveillance of current changes in the epidemiology of *Invasive Fungal Diseases* (IFDs) as an important component of the antifungal stewardship programs (ASP), requires careful regular monitoring, especially in high-risk settings such as oncology centers. This study aimed to examine *Candida* colonization status and corresponding current changes in children with malignancy during repeated admissions and also investigate the possible epidemiological shifts after the implementation of ASP.

**Methods:**

In this prospective observational study, all eligible patients younger than 18 years were recruited during 2016–2017 at *Amir Medical Oncology Center* (AMOC) in Shiraz, Iran. Totally, 136 patients were enrolled and 482 samples were collected from different sites (oral/nasal discharges, urine and stool). Weekly regular sampling was carried out during hospitalization. *Candida* colonization status and epidemiological changes were monitored during repeated admissions. Samples were cultivated on Sabouraud Dextrose agar medium and identified by Polymerase Chain Reaction -Restriction Fragment Length Polymorphism (PCR-RFLP).

**Results:**

Estimated *Candida* colonization incidence was 59.9% (82/136) in our patients. *Candida* colonization was found to be higher in oral cavity and rectum than that in nasal cavity. Among those long-term follow ups and repetitive hospitalizations, a significant number of patients exhibited changes in their colonization patterns (37.7%). *Candida* colonization did not reveal any significant relationship with age, sex, oncologic diseases and degree of neutropenia. *C. albicans* (72.0%) was found as the most common *Candida* species in colonized patients, followed by *C. krusei*, *C. kefyr*, *C. glabrata* and *C. parapsilosis*.

**Conclusion:**

Given the high incidence of *Candida* infections in children with cancers, close monitoring of epidemiologic changes is essential for judicious management, based on local surveillance data and improvement of overall quality of care in high risk patients.

## Background

Prevention and management of invasive fungal diseases (IFDs) may be one of the most challenging problems in children suffering from cancer. Despite recent improvements in diagnosis and management of IFDs, we still witness high attributable morbidity and mortality in those with hematological malignancies [1, 2]. Among various yeast and mold infections in immunocompromised hosts, *Candida* is the most common cause with wide spectrum clinical manifestations ranging from local infection to severe multi-organ involvement [3]. *Candida* colonization in hematological malignancies could affect final clinical outcome, especially when colonization occurs with a strain that has reduced susceptibility to azole antifungals [4, 5]. Also, with prolonged colonization during chemotherapy, the possibility of invasive candidiasis increases with the same colonized isolates [6]. Although *C. albicans* literally is considered as the leading cause of all *Candida* related IFDs, a shift toward *non-albicans* infections has been identified globally [7–9]. Among the various types of *non-albicans* species, there is growing concern about some of them such as *C. glabrata* and *C. parapsilosis* because of varying degrees of *Azole* antifungal resistance [3, 10]. Changes in colonization may happen during repeated admissions and various risk factors may make children with hematological malignancies prone to new colonization (especially resistance ones), multi-species colonization or changed primary colonization [11]. Some of these possible risk factors may be broad-spectrum antibiotic therapy, antifungal prophylaxis, *Azole* antifungal misuse, various types of short/long term indwelling catheters, total parenteral nutrition, neutropenia, surgical interventions, changes in chemotherapy intensity due to refractory disease or relapse, younger age at oncologic diagnosis, repeated IFDs during course of treatment and comorbidities such as hyperglycemia [11–15]. Some of these variables are known risk factor for *Candida* colonization while the role of others needs to be determined by further risk studies [16]. Although attention to these risk factors and application of proper infection prevention strategies for controllable ones (prevention of *Azole* antifungal misuse, implementation of antimicrobial stewardship programs and prevention of unnecessary prolong catheterization) is almost always ineluctable [17], such changes in *Candida* colonization should be documented primarily in each individual during repeated admissions by frequent sampling from different possible colonization sites. This study seeks to investigate the epidemiology of *Candida* colonization and any possible changes in colonization pattern in children with malignancies as a part of regular local surveillance program in AMOC in Shiraz, Iran. Indeed, the study was conducted in the continuation of the previous research carried out in 2011-2012 by Haddadi et al. [18] to investigate the epidemiology of candida colonization and its possible changes during recurrent admissions in AMOC.

## Methods

This prospective, observational study was performed during 2016–2017 on the children hospitalized in a referral oncology teaching hospital, Shiraz, Iran. Amir Hospital is an oncology hospital with 54 beds for children and about 5000 admitted children and adolescents under 18 years of age per each year. This study was conducted on the pediatric patients under 18 years who were suffering from hematological malignancies or solid organ tumors. The study procedure was started after filling consent forms by the patients’ parents. As an observational study, demographic data were obtained using predesigned questionnaires.

To examine the rate of *Candida* colonization, oral, urine, nasal, and stool samples were collected upon admission in the wards. Only urine and stool samples were collected from those with severe thrombocytopenia or any type of bleeding tendency and oral and nasal samples were withheld in such cases. To observe the changes in colonization pattern during hospitalization, weekly sampling was performed during total course of admission. The *Candida* colonization index (CI), was calculated as the ratio of the number of non-blood body sites colonized by Candida spp. to the total number of body sites cultured. Samples were cultivated on Sabouraud Dextrose Agar Medium (Merck, Germany). The cultivated Sabouraud Dextrose Agar plates were incubated in ambient temperature and then, the species were identified at mycology division of *Professor Alborzi Clinical Microbiology Research Center* (PACMRC). *Candida* spp. were identified by the formation of germ tube and PCR-RFLP (Polymerase Chain Reaction -Restriction Fragment Length Polymorphism) using forward ITSI1 (5′-TTCCGTAGGTGAACCTGCGG-3′) and reverse ITS4 (5′-TCCTCCGCTTATTGATATGC-3′) primers. The products were digested using MspI (#ER0541) restriction enzyme [19]. The normal distribution was confirmed by Kolmogorov–Smirnov test and the obtained data were analyzed by independent sample T-test, Chi-square test using SPSS version 21.0 (IBM Corp. IBM SPSS Statistics for Windows, Armonk, NY). The significance level (*P*-value) of all tests was below 0.05 and α = 0.05. Comparison of the proportions between the present study and our recent study in 2011–2012, was done using MedCalc Software bvba, Version 18.9.1.

## Results

### Demographics

**Fig. 1:**
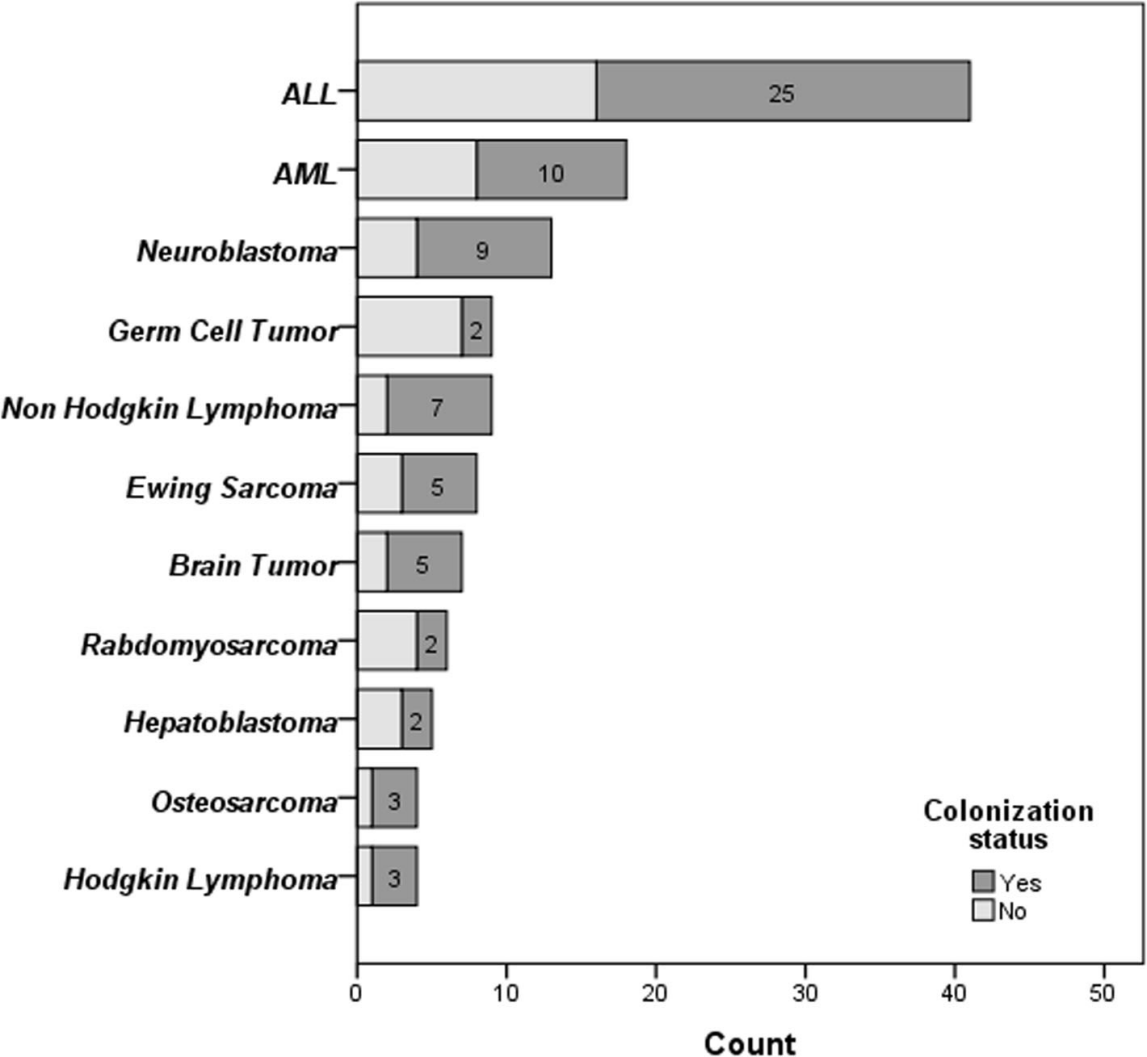
*Candida* colonization status in children with different malignancies. Data are shown based on the most common oncologic diagnosis (data presented in number). Difference was not significant (see text for more information)

After exclusion, 136 pediatric patients younger than 18 years were recruited in the study and totally, 482 samples were collected from different sites (oral/nasal discharges, urine and stool). Among them 82 (59.9%) were male. The average age was calculated 7.1 years-old (6 ± 4.69 SD, 4 and 18, minimum and maximum, respectively). Detailed data about sex and patient’s age category are shown in Table 1. As for the primary underlying disease, *Acute Lymphoblastic Leukemia* (ALL) was the most common oncologic diagnosis (41, 30.1%). Close to 85% (115/136) of the patients had history of recurrent admission (Table 1). The colonization rate was greater in the patients with history of recurrent admission (61.7% versus 38.3%). Totally, 39% of patients had long term follow ups. Of special note, among children with long term follow ups, 71.7% were colonized. In this subgroup of patients, repeated admission was found to be correlated with greater chance of *Candida* colonization (*P*-value = 0.014). Common oncological diagnosis and also their colonization status have been shown in Fig. 1.

**Table 1:** Demographic data in colonized and non- colonized children with malignancy

		Colonization status				P. value^a^
		Yes		No		
		Count	%	Count	%	
Gender	Female	35	63.6%	20	36.4%	.512
	Male	47	58.0%	34	42.0%	
Age group	≤12 months	0	0.0%	3	100.0%	
	1–5 years	29	59.2%	20	40.8%	.088
	> 5 years	53	63.1%	31	36.9%	
Neutropenia (ANC < 1500)	Yes	46	65.7%	24	34.3%	.160
	No	35	53.8%	30	46.2%	
History of admission	First admission	11	52.4%	10	47.6%	.420
	Recurrent admission	71	61.7%	44	38.3%	

a. P-value by Fisher’s Exact Test

### Samples’ characteristics

Four hundred and eighty-two samples were collected from different sites and finally 140 positive samples were analyzed (excluding negative cultures). About 80% of the patients underwent at least two-time sampling (based on weekly sampling protocol) and more than 20% underwent 3 times or more. Among the collected samples, 195 (40%) were from oral cavity, 220 (46%) from nasal discharges, 44 (9%) from urine, and 24 (5%) from stool. There was no significant difference between colonized and non-colonized patients with regard to age category and sex (P-value = 0.088 and 0.593, respectively).

### Colonization status

#### A. Patient’s colonization status

*Candida* colonization was found in 59.9% (82/136) of the patients. Most children exhibited oral colonization (67.1%). Oral/Rectal colonization (11%) was the second most common type of colonization among the studied children (Table 2). 72% of the children were colonized with *C. albicans*, followed by *C. krusei* (9.8%), *C. kefyr* (7.3%), *C. glabrata* (2.4%) and *C. parapsilosis* (2.4%). Among the studied patients, one had double colonization with *C. kefir* and *C. parapsilosis* and another one with *C. albicans* and *C. tropicalis*. Data regarding distribution of different colonization sites in colonized children are presented in Table 2. Calculated mean Candida CI was 0.311 ± 0.121 [1:4 (78%), 2:4 (19.5%) and 3:4 (2.4%)]. Colonization site preferences were analyzed by Chi-Square Tests for age, sex, degree of neutropenia, primary oncologic diagnosis and recurrent versus new admission (Table 3), revealing no significant differences (*p* = 0.531, *p* = 0.304, 0.125, 0.551 and 0.797, respectively). Also, Candida CI was investigated for these variables that revealed no significant differences, either (*p* = 0.650, *p* = 0.246, 0.259, 0.701 and 0.307, respectively).

**Table 2:** Distribution of different colonization sites in 82 colonized children with malignancy ^a^

		Frequency	Valid Percent	Cumulative Percent	
	Oral	55	67.1	67.1	
	Nasal	3	3.7	70.7	
	Fecal	6	7.3	78.0	
	Oral/Nasal	6	7.3	85.4	
	Oral/Rectal	9	11.0	96.3	
	Nasal/ Rectal	1	1.2	97.6	
	Oral/Nasal/ Rectal	2	2.4	100.0	
	Total	82	100.0		

a. Urinary colonization was not found

**Table 3:** Incidence of different *Candida* species in males and females, predefined age groups, neutropenic patients and those with short and long term follow up

Species type	Sex				Age group*				Neutropenia				Follow up duration						
	Female		Male		1–5 year		> 5 year		Yes		No		No		Short term		Long term		
	Count	%	Count	%	Count	%	Count	%	Count	%	Count	%	Count	%	Count	%	Count	%	
*C. albicans*	23	65.7%	36	76.6%	20	33.9%	39	66.1%	34	58.6%	24	41.4%	31	52.5%	2	3.4%	26	44.1%	
*C. krusei*	1	2.9%	5	10.6%	1	16.7%	5	83.3%	4	66.7%	2	33.3%	3	50.0%	1	16.7%	2	33.3%	
C. Kefyr	1	2.9%	1	2.1%	1	50.0%	1	50.0%	2	100.0%	0	0.0%	0	0.0%	0	0.0%	2	100.0%	
*C. glabrata*	1	2.9%	1	2.1%	2	100.0%	0	0.0%	0	0.0%	2	100.0%	0	0.0%	0	0.0%	2	100.0%	
*C. parapsilosis*	0	0.0%	2	4.3%	1	50.0%	1	50.0%	1	50.0%	1	50.0%	1	50.0%	0	0.0%	1	50.0%	
*C. lusitaniae*	1	2.9%	0	0.0%	0	0.0%	1	100.0%	0	0.0%	1	100.0%	0	0.0%	0	0.0%	1	100.0%	
C. Albicans/*C. tropicalis*	0	0.0%	1	2.1%	0	0.0%	1	100.0%	0	0.0%	1	100.0%	1	100.0%	0	0.0%	0	0.0%	
C. Krusei/C. Tropicalis	0	0.0%	0	0.0%	0	0.0%	0	0.0%	0	0.0%	0	0.0%	0	0.0%	0	0.0%	0	0.0%	
C. Kefyr/C. Parapsilosis	0	0.0%	1	2.1%	0	0.0%	1	100.0%	1	100.0%	0	0.0%	0	0.0%	1	100.0%	0	0.0%	
Not defined	8	22.9%	0	0.0%	4	50.0%	4	50.0%	4	50.0%	4	50.0%	4	50.0%	0	0.0%	4	50.0%	

*Colonization was not found in children younger than 12 months of age in this study

#### B. Sample’s characteristics

**Fig. 2:**
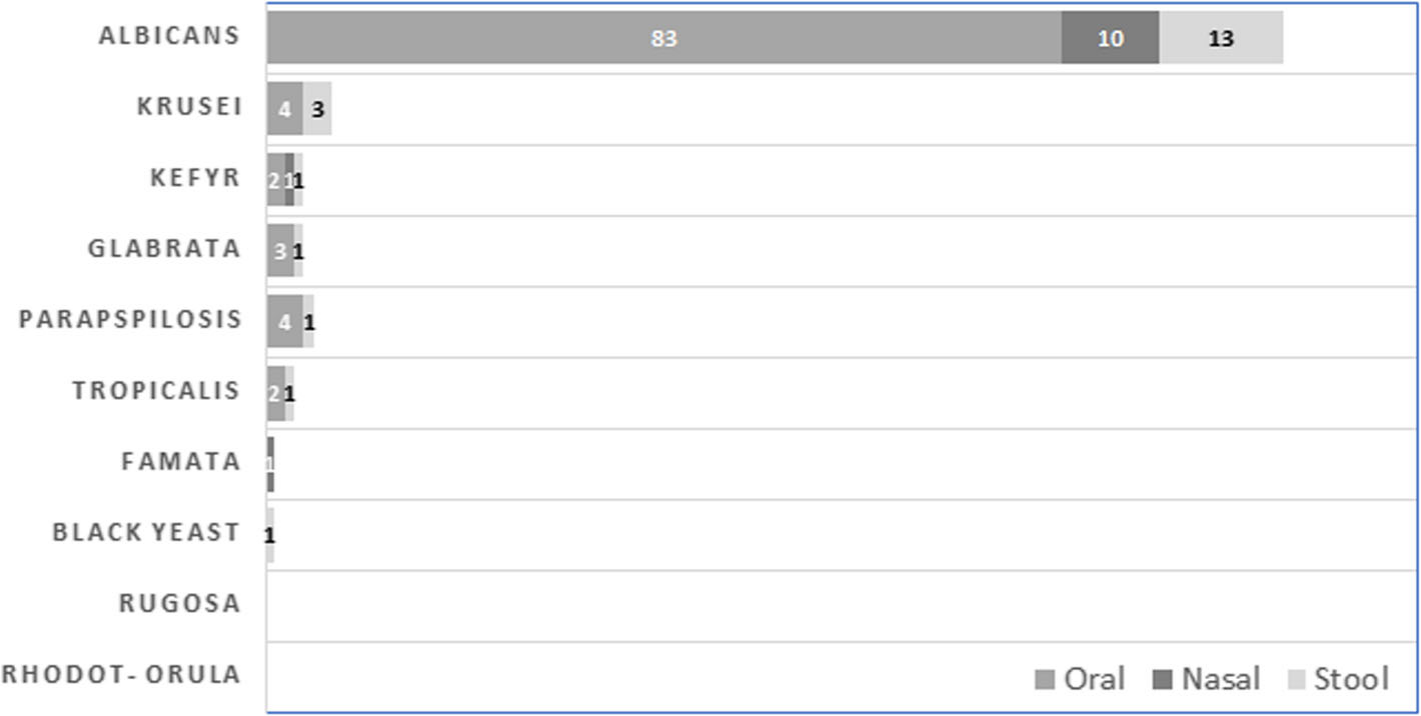
Distribution of different *Candida* spp. in oral cavity, nasal cavity and rectum among colonized patients

From 140 positive samples (excluding black yeasts), species type was determined in 130 samples (10 samples were not recognized). Oral cavity was the most common site of colonization (Fig. 2), followed by nasal cavity and rectum, 98 (75.4%)**,** 12 (9.2%) and 20 (15.4%), respectively (calculated from all positive samples). Of special note, in this study we did not find any urinary candida colonization among the studied children. Regarding sex differences, we did not observe any correlation between colonization status and sex (*P*-value = 0.593). In terms of age distribution, most of colonized children were over 5 years (53, 63.1%) and the rest 1–5 years (29, 59.2%). Although colonization rate was directly correlated with age category (greater in patients > 5 years), the difference was not significant (*P*-value = 0.088, LR: 0.05). *Candida* colonization status was investigated based on different underlying oncologic diagnoses. ALL and AML were the most common diagnoses in both colonized and non-colonized children without any significant difference (*P*-value = 0.432). No statistically significant relationship was found between *Candida* colonization and the severity of neutropenia during admission (*P*-value = 0.166).

#### *C. candida* species characteristics

**Fig. 3:**
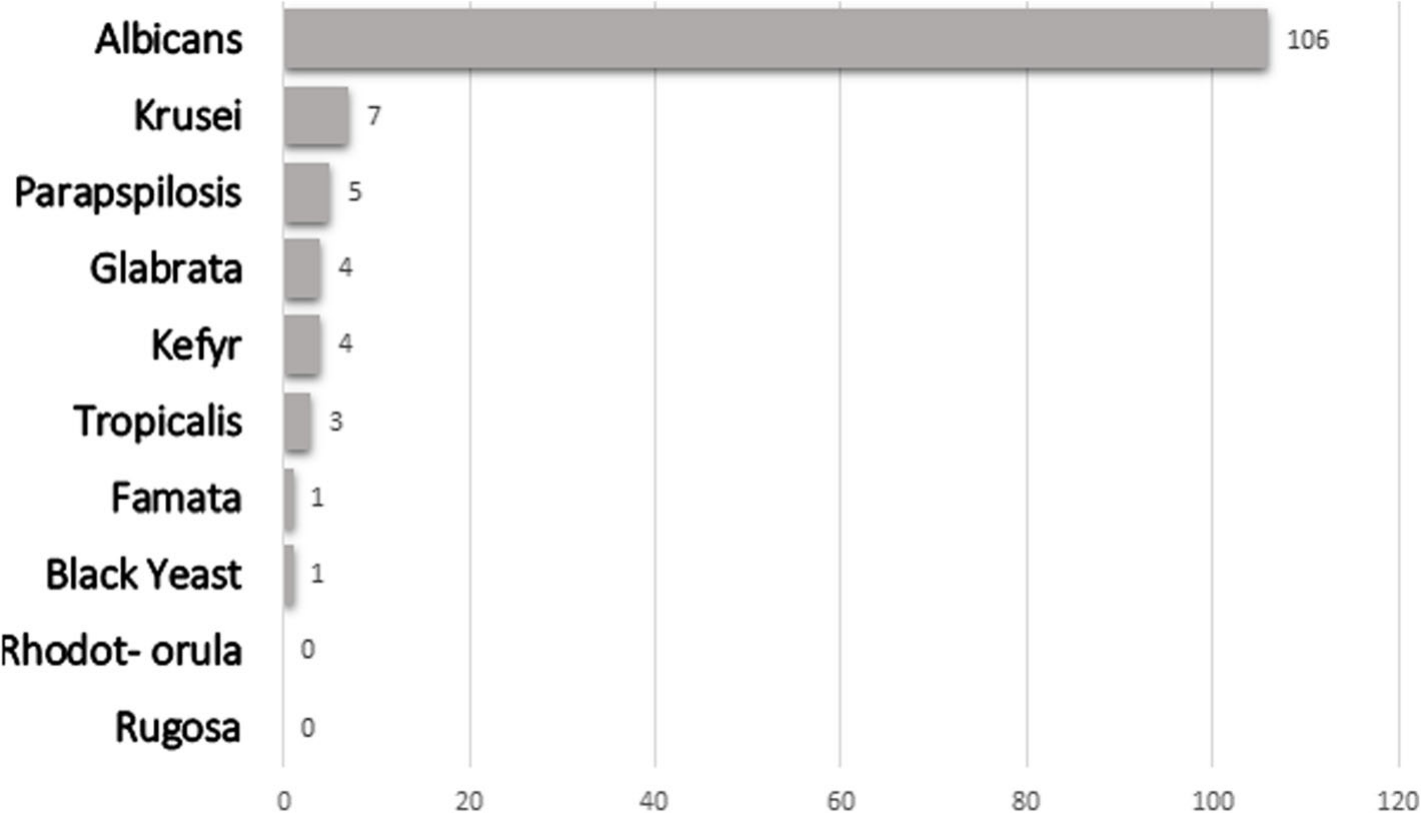
Different *Candida* spp. which were recognized in colonized patients (among 131 identified samples including black yeast)

Among the different types of recognized *Candida* species (140 spp.), *C. albicans* was the most common (72%), followed by *C. krusei* (9.8%), *C. kefyr* (7.3%), *C. glabrata* (2.4%) and *C. parapsilosis* (2.4%); data shown in Fig. 3. Of the studied patients, one had double colonization with *C. kefir* and *C. parapsilosis* and another with *C. albicans* and *C. tropicalis*. A small proportion of the species could not be distinguished phenotypically on culture media and was categorized as “Not defined” (10 samples). Detailed data regarding different *Candida* species in oral and nasal discharges, and stool samples are summarized in Table 4.

**Table 4:** The prevalence of colonization with different *Candida* species in oral and nasal discharges, and stool and urine samples

Total samples (number)	Candida species	Number of positive samples	Prevalence (% of total oral samples)
Oral (195)	*C. Albicans*	83	77.6
	*C. Krusei*	4	3.7
	*C. Parapsilosis*	4	3.7
	*C. Glabrata*	3	2.8
	*C. Kefyr*	2	1.8
	*C. Tropicalis*	2	1.8
	Not defined	9	8.4
Nasal (220)	*C. Albicans*	10	76.9
	*C. kefir*	1	7.6
	*C. famata*	1	7.6
	Not defined	1	7.6
Stool (24)	*C. Albicans*	13	65
	*C. Krusei*	3	15
	*C. Kefyr*	1	5
	*C. Parapsilosis*	1	5
	*C. Glabrata*	1	5
	*C. Tropicalis*	1	5
Urine (44) *	–	–	–

*No colonization was found in urine specimens

#### D. Monitoring of colonization pattern during patient’s follow ups

On average, 53 cases were followed for at least 4 weeks (26.87 days ±SD, 39.6). Follow up duration also was categorized in four-time frame as < 30 days, 30–60 days, 60–90 days and > 90 days. More than 64% of colonized children were followed for more than 4 weeks (Table 5).

**Table 5:** Follow-up time frames in colonized children with malignancy

		Count	%	Cumulative Percent
Follow-up time frames	> 90 days	14	26.4	26.4
	60–90 days	11	20.7	47.1
	30–60 days	9	16.9	64.2
	< 30 days	19	35.8	100
	Total	53	100	

During the study period, 6.1% of monitored cases had changes in their colonization patterns, indicating non-colonization to colonization and vice versa and also change in candida species (Table 6). During repeated sampling, in 2.2% of colonized cases, colonization was not continued (that indicates negative culture on two occasions at least two weeks apart).

**Table 6:** Significant changes in colonization status during follow-up

	Frequency	Percent ^a^	Valid Percent ^b^	Cumulative Percent
“No colonization” to “colonization”	10	7.4	50.0	50.0
Change to “colonization with other candida species”	7	5.1	35.0	85.0
“Decolonization” ^c^	3	2.2	15.0	100.0
Total	20	14.7	100.0	

a. From total colonized cases

b. Calculated from those in whom colonization change were detected

c. Negative culture on two occasions at least two weeks apart

The association between recurrent admissions and any type of colonization status change was assessed by Chi-Square Tests which revealed a significant correlation (*p* = 0.035).

## Discussion

Without systematic use of antifungal prophylaxis, invasive *Candida* infections account for the most prevalent type of IFDs in onco-hematological centers [14, 18, 20] and usually is considered as a consequence of primary colonization [17]. In this study, nearly 60% of all investigated children were colonized with different *Candida* species. Compared to our recent study in 2011–2012 [18], we found important findings which needs special consideration.

First, compared to the previous study, we found a substantial increase in colonization rate (46.5% versus 59.9%) [Difference 13.4, 95% CI: 2.39 to 23.91%, *P* = 0.0173]. In addition to possibly true increases in the incidence of *Candida* colonization, this increase also could be partially due to regular sampling program which was conducted by the main investigator during the study period.

Second, we found that *Candida non-Albicans* colonization rate decreased significantly from 38 to 18.5% (*P* = 0.0003). Although antifungal prophylaxis in onco-hematological patients receiving intensive chemotherapy is encouraged based on logical argument for reducing mucosal colonization and consequently systemic invasive disease, prolonged azole exposure during antifungal prophylaxis also is considered an important risk factor for shifting in epidemiology of candidiasis [20, 21]. Due to the very low incidence of proven and probable invasive *Candida* infections in AMOC, currently (after October 2015) Azole prophylaxis is not routinely used in pediatric patients with hematological malignancies as a part of antifungal stewardship program (ASP). Along with other reports this strategy may explain such a decrease in *Candida non-Albicans* colonization rate despite a total increase in the incidence of *Candida* colonization and emphasis on the role of ASP in controlling undesired changing in the epidemiology of candidiasis [21, 22].

Third, similar to our previous report, *C. krusei* was the most prevalent type of *non-Albicans* species in colonized children. As known, *C. krusei* usually is considered as an important Azole-resistant *non-Albicans* colonizer with a high MIC to fluconazole and voriconazole [10].

Fourth, we found some increase in the prevalence of *C. parapsilosis* and conversely significant decrease in *C. famata* prevalence in this study. *C. parapsilosis* was the second most common *non-Albicans* species in colonized children, just after *C. krusei*, whereas in our previous study *C. glabrata*, *C. tropicalis* and *C. famata* were more prevalent than *C. parapsilosis*. This is of utmost importance because increased resistance to Azoles frequently was seen with *C. parapsilosis* [23]. Corresponding data regarding common *non-Albicans* species were shown in Table 7.

**Table 7:** Colonization characteristics in both studies conducted in AMOC, in children who were colonized with different Candida species

	2017–2018		2011–2012 (18)		*p*-value ^c^
	N ^b^	%	N ^b^	%	
Candida species ^a^					
C. *Albicans*	106	81.6	117	62	0.0003*
C. *Krusei*	7	5.4	18	9.6	0.2477
C. *Parapsilosis*	5	3.9	8	4.3	0.9126
C. *Glabrata*	4	3.1	14	7.5	0.1559
C. *Kefyr*	4	3.1	4	2.2	0.8893
C. *Tropicalis*	3	2.4	11	5.9	0.2262
C. *Famata*	1	0.8	11	5.9	0.0416*
Albicans versus non-Albicans Candida species					
Albicans	106	81.6	117	62	0.0003*
Non-Albicans	24	18.5	72	38	0.0003*
Colonization site					
Oral	98	75.4	88	38.4	< 0.0001*
Nasal	12	9.2	24	10.4	0.8560
Rectal	20	15.4	77	33.6	0.0003*
Urine	0	0	40	17.4	< 0.0001*

a. Very rare species including *C. guilliermondii*, *C. rugosa*, *C. Lusitaniae*, *C. lambica*, and *Rhodotorula spp*. were excluded from 2011 to 2012 study

b. Number of positive samples

c. Chi-square test

*Statistically significant

Comparable to the previous study in AMOC, ALL was found as the most common oncologic diagnosis in children with hematological malignancies. While acute leukemia was the most common diagnosis in different similar studies, the leukemia type may vary, based on different geographic regions [6, 16]. In contrast to the study by Albert et al. [24], we did not find any relationship between colonization status or its change with neutropenia. Treatment of newly diagnosed standard risk ALL usually leads to the first successful remission. Excluding a small number of patients with acceptable response to induction chemotherapy, others usually undergo consequent phases of treatment leading to prolonged maintenance treatment phase which is based on the administration of less intensive regimen on an outpatient basis [25]. There are several causes that lead to repeated admissions for children with hematological malignancies and such children have greater hospitalization rates, (more than 2-folds), compared to the general population [26]. The possible role of recurrent hospitalization on *Candida* colonization pattern in children with hematological malignancies has not been determined exactly and in this study, we decided to address this issue. More than two-thirds of our studied cases were on maintenance chemotherapy with recurrent admissions. Although *Candida* colonization rate was significantly greater (more than 6:1) in patients with history of recurrent admission, difference was not statistically significant (*p* = 0.420). However, we found that in the subgroup of children with long term follow up, repeated admission was significantly correlated with a higher *Candida* colonization rate. Similar to other reports [27], oral cavity was the most common *Candida* colonization site both in this study and our previous report in 2014 [18], but we found some considerable changes in the present study. First, compared to the previous study, the rate of oral colonization significantly increased (75.4% versus 38.4%). Secondly, we did not find any urinary colonization in this study (Table 6). This may be due to successful adherence to AFS (avoidance of unnecessary catheterization, timely removal of unnecessary catheters and last but not least implementation of antimicrobial stewardship program in our center).

Finally, reports on epidemiologic changes in *Candida* colonization during repeated admissions are somewhat lacking in the literature, especially in children with malignancies [28]. Our study confirmed that repeated hospitalization in children with malignancy (especially those with ALL) has an important role in changing the face of *Candida* colonization. Consistent with the study by Kaben et al. [29], we observed some changes to *non-Albicans* species during hospitalization (5.1%, Table 5). Despite observed changes in the pattern of candida colonization and also implementation of non-Azole prophylaxis in high-risk patients, it should be noted that no significant changes have occurred to the burden of candidemia. Incidence rates of candidemia were 4.3, 4.1 and 4.5% during 2015, 2016 and 2017, respectively.

### Limitations

This study has some limitations. We encountered inevitable limitations in regular and timely sampling from all pre-defined sites due to uncontrolled patients’ clinical status. Among them we could mention increased bleeding tendency, thrombocytopenia, delay in taking urine and stool samples in young infants.

## Conclusion

Changing face of *Candida* colonization pattern among high-risk colonized children with different types of malignancies may warrant judicious use of Azoles agents and an urgent need for the implementation of ASP to reduce colonization of resistant *Candida* spp.

## Abbreviations

AMOC: Amir Medical Oncology Center
ASP: Antifungal Stewardship Program
CI: Colonization Index
IFDs: Invasive Fungal Diseases
PACMRC: Professor Alborzi clinical microbiology research center
